# Redescription of the Dikraneurini leafhopper *Dikrellamella* Ruppel & DeLong, 1952 (Hemiptera, Cicadellidae) with a synoptic checklist of leafhoppers on avocado trees in Mexico

**DOI:** 10.3897/zookeys.857.33910

**Published:** 2019-06-24

**Authors:** J. Adilson Pinedo-Escatel, Dmitry Dmitriev

**Affiliations:** 1 Departamento de Botánica y Zoología, CUCBA, Universidad de Guadalajara, km 15.5 carretera Guadalajara-Nogales, Las Agujas, Zapopan, C.P. 45110, Apdo. Postal 139, Jalisco, México Universidad de Guadalajara Zapopan Mexico; 2 Prairie Research Institute, 2204 Griffith Dr., Building 11 Dock A, Champaign, IL 61820, USA Prairie Research Institute Champaign United States of America

**Keywords:** *
Alconeura
*, Auchenorrhyncha, *
Empoasca
*, Empoascini, *
Idona
*, *
Joruma
*, Typhlocybinae

## Abstract

Among leafhoppers (Hemiptera, Cicadellidae), only Typhlocybinae are known in Mexico to inhabit avocado, an important horticultural crop. In this paper, a potential avocado pest, *Dikrellamella* Ruppel & DeLong, 1952, is redescribed and illustrated. Additionally, a detailed checklist and a key for all known species of Typhlocybinae associated with avocado trees in Mexico are provided.

## Introduction

Herbivorous sap-sucking insects are potentially devastating agricultural pests because they not only injure plants directly but may also transmit plant pathogens (Bosco and Marzachi 2016). Most such pests belong to the order Hemiptera (Hogenhout et al. 2008), of which the family Cicadellidae (leafhoppers) (Hemiptera: Auchenorrhyncha) is the most relevant because it comprises around 75% of plant pathogen vector species (Weintraub and Beanland 2006). Within leafhoppers, the subfamily Typhlocybinae is reported to spread several kinds of pathogens effectively due to their high capacity for dispersal. Leafhopper vectors often go unnoticed when transmitting plant diseases, their presence only being detected after disease outbreaks occur (Nault 1979).

Avocado is one of the most important horticultural crops worldwide and Mexico is the main exporter (SAGARPA 2017). Recently five species of leafhoppers were identified as being associated with avocado trees in central Mexico (Quezada-Daniel et al. 2017). All of those species belong to the subfamily Typhlocybinae. Our study of leafhoppers from several entomological collections in Mexico revealed that these species have been widely misidentified. For example, specimens of *Dikrellamella* Ruppel & DeLong, 1952, housed in Mexican collections were often misidentified as *Empoasca* spp., presumably based on superficial resemblance in size and coloration.

The genus *Dikrella* Oman, 1949 was described based on type-species *Dikraneuracockerellii* Gillette, 1895. Oman (1949) also moved 14 species previously placed in *Dikraneura* Hardy, 1850 to *Dikrella*. Today, the genus includes two subgenera: *Readionia* Young, 1952 with four species and *Dikrella* Oman, 1949 with 37 well-defined species and three subspecies. The genus is restricted to the New World. Only one species of the genus is known so far to be a potential vector of diseases of avocado crops.

*Dikrellamella* Ruppel & DeLong, 1952 was described from four localities in Mexico based on two males and four females. The original description and illustrations lack important details useful for distinguishing the species. Since then, no further information was published on its distribution or host plants. Here we provide a redescription and diagnostic illustrations of this important avocado leafhopper. We also provide a detailed checklist and a key to all known species recorded from avocado trees in Mexico.

## Materials and methods

All specimens identified in this study are housed at the Colección Nacional de Insectos, Instituto de Biología, Universidad Nacional Autónoma de México, Mexico (**CNIN**), Colección de Insectos del Instituto de Fitosanidad, Colegio de Postgraduados, Texcoco, Estado de México, Mexico (**CEAM**), Colección de Auchenorrhyncha de Jorge Adilson Pinedo Escatel, Mexico (**CAJAPE**), Colección Entomológica del Centro de Estudios en Zoología, Universidad de Guadalajara, Zapopan, Mexico (**CZUG**), and C.A. Triplehorn Insect Collection, Ohio State University, Columbus, USA (**OSUC**).

Taxonomic criteria and terminology follows mainly Young (1952), Dietrich (2005), and Dmitriev (2010). Techniques for preparation of male genital structures follow Oman (1949) modified such that male abdomens were rinsed with water mixed with alcohol at different concentrations. Label data are given between quotation marks, with a backslash (\) separating the lines on the labels. Images of habitus were taken using a Carl Zeiss camera mounted on a Stemi 2000c stereo-microscope, and illustrations were drawn using a camera lucida attached to a Leica stereo microscope. Subsequently, drawings were digitized and vectorized with Adobe Illustrator and edited in Adobe Photoshop. Measurements were obtained using an electronic vernier.

## Taxonomy

### Typhlocybinae Kirschbaum, 1868

#### Dikraneurini McAtee, 1926

##### Dikrella

Taxon classificationAnimaliaHemipteraCicadellidae

Oman, 1949

Dikrella (Dikrella) Oman, 1949
Dikrella (Dikrella) Oman, 1949: 83.

###### Type-species.

*Dikraneuracockerellii* Gillette, 1895

###### Diagnosis.

Slender leafhoppers, overall body coloration usually white to yellowish. Head as wide as pronotum, produced, crown convex. Forewing fourth apical cell short and third narrow. Hind wing submarginal vein complete, three apical cells. Pygofer with process. Aedeagus body elongate or robust usually with a pair of basal process.

###### Remarks.

*Dikrella* differs from *Kunzeana* Oman, 1940 by the distinctly widened basal part of the forewing inner apical cell.

###### Distribution.

Confined to the New World, recorded from: United States, Mexico, Costa Rica, Cuba, Puerto Rico, Panama, Canada, Ecuador, Colombia, Bolivia, and Brazil.

##### Dikrella (Dikrella) mella

Taxon classificationAnimaliaHemipteraCicadellidae

Ruppel & DeLong, 1952

Figures 1 , 2 , 3–8

Dikrella (Dikrella) mella Ruppel & DeLong, 1952: 90

###### Description of male.

**Figure 1.**
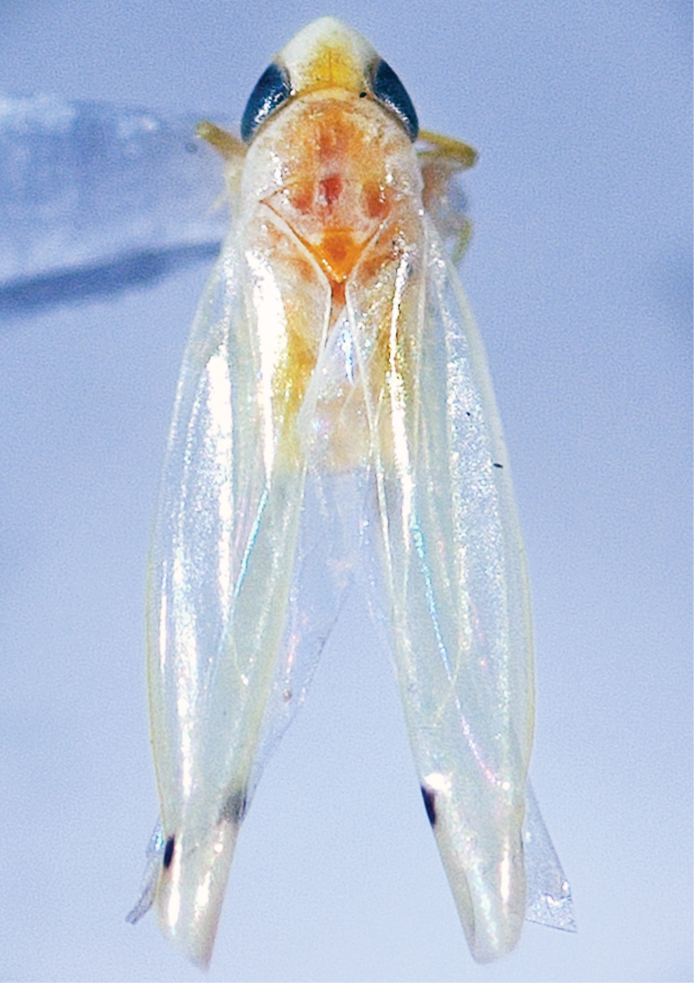
*Dikrellamella* Ruppel & DeLong, 1952 male body, dorsal aspect.

**Figure 2.**
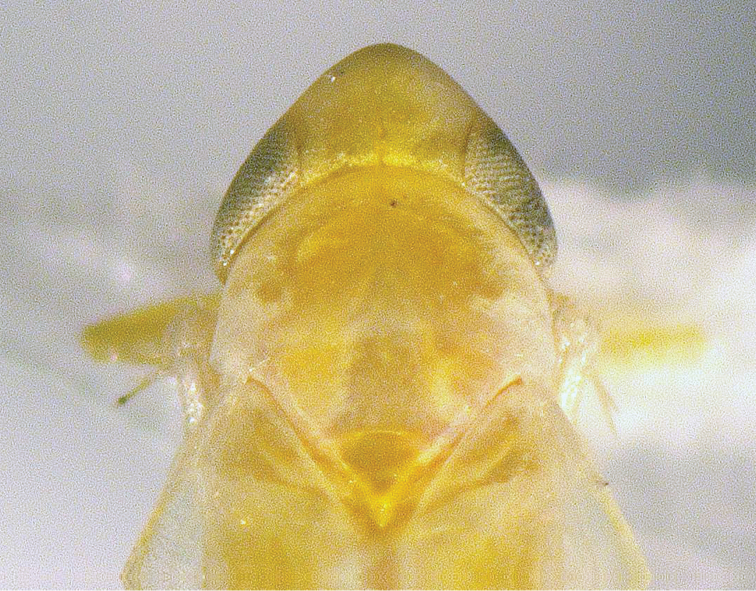
*Dikrellamella* Ruppel & DeLong, 1952 female head, dorsal aspect.

Small, delicate. Body slender. Texture of head, pronotum, and mesonotum uniform. General coloration yellowish with orange-gold infusions on pronotum and ventral view, forewing with two black spots on first and fourth apical cell, spots of same diameter but one in fourth cell lighter (Figs 1 and 2). Head well produced, narrowly rounded apically, lateral margin white, center yellow, distance between eyes (interocular) 1.0 × of eye diameter, coronal suture half as long as crown length. Face without marks, mostly white-yellowish. Frontoclypeus narrow and parallel-sided. Anteclypeus longer than wide. Pronotum large, produced anteriorly, reaching half-length of eye, convex, slightly wider than head, lateral margins slightly convergent distally, white, center yellow. Visible part of mesonotum large, as long as pronotum, apex gold. Forewing well developed, translucent with tiny yellow marks along sides of R, M veins, and apical cells, some yellow pigmentation at base of marginal vein and clavus. Hind wing translucent.

###### Description of female.

Same as male but color somewhat paler overall.

###### Male genitalia.

**Figure 3–8.**
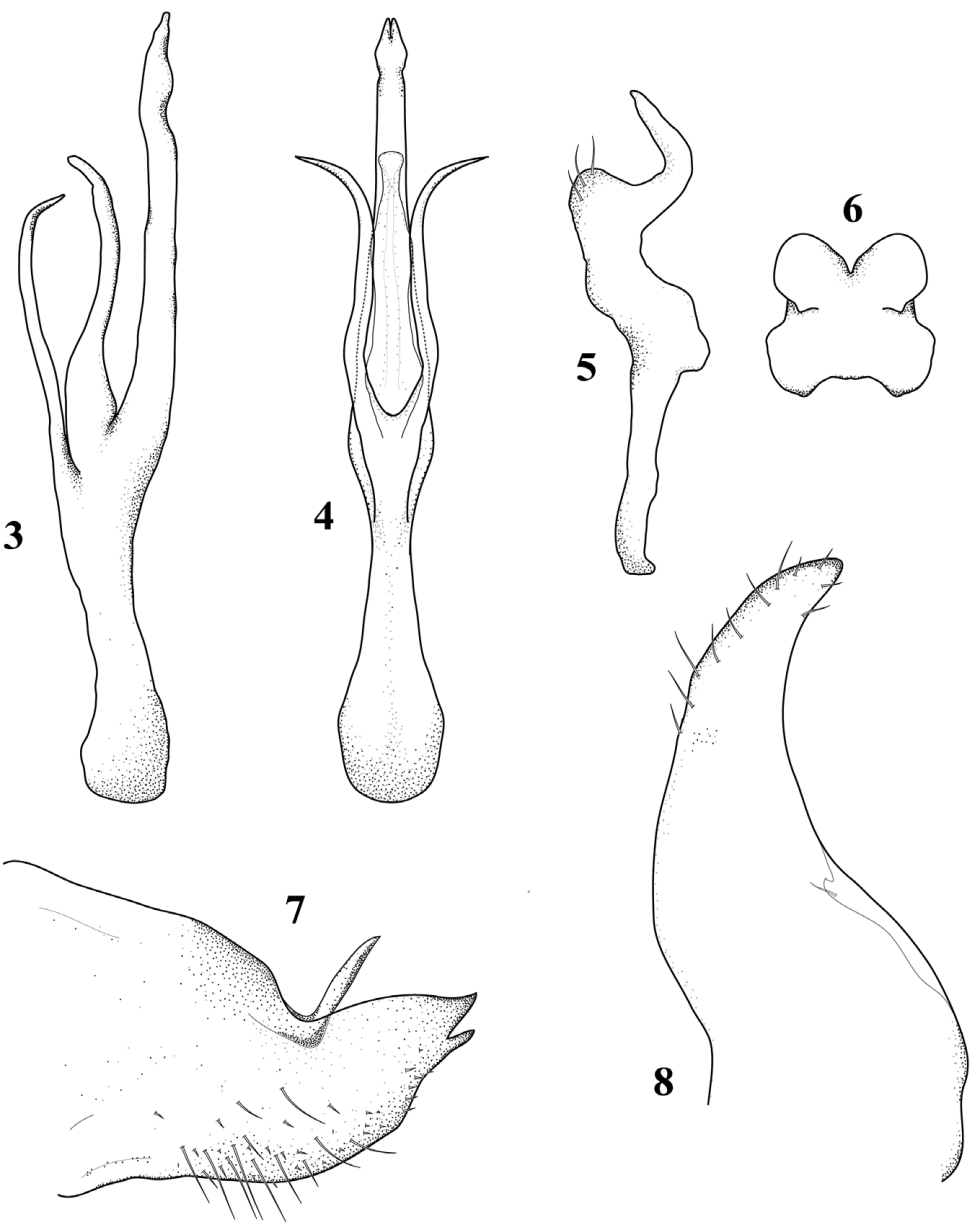
*Dikrellamella* Ruppel & DeLong, 1952 male genitalia: **3** aedeagus, lateral view **4** aedeagus, ventral view **5** style, dorsal view **6** connective, dorsal view **7** pygofer, lateral view **8** plate, dorsal view.

Pygofer conical, narrowing caudally, longer than wide, with notch on dorsal margin, dorsal process slender curved dorsad arising beyond midlength of pygofer near dorsal notch; ventral process short, straight subapical (Fig. 7). Anal tube broad and membranous. Subgenital plate elongate, wider at base and narrowed toward tip, apex rounded, outer margin striate, inconspicuous setae running on each side of plate (Fig. 8). Connective broad and short, almost square (Fig. 6). Style long, base narrow, anterior lobe not developed, preapical lobe very bulky, projected laterad with fine setae apically; apex long, curved and finger-shaped (Fig. 5). Aedeagus with atrium about as long as shaft, dorsal apodeme not developed; shaft long, slender and slightly curved dorsad with dorsal preapical gonopore; ventral appendage large, forked close to apex, straight in lateral view; atrium with two long slender processes arising near base of shaft, parallel to each other on ventral side of shaft, divergent at apex (Figs 3 and 4).

###### Female genitalia.

VII sternite quadrate, ovipositor large, pointed.

###### Immature stages.

Unknown

###### Measurements.

Body size 3.0–3.5 mm

###### Reported hosts.

Avocado (*Perseaamericana*)

###### Type locality.

Cuernavaca, Morelos state (Mexico)

###### Distribution.

Mexico: Guerrero (Iguala), Michoacán (Uruapan), Oaxaca (Rancho Monter), Morelos (Cuernavaca; Tetela del Volcán), and Chiapas (Vergel)

###### Material examined.

Holotype ♂ (OSUC), MEXICO: Cuernavaca Mor. \ X-21-41. \ K. 57 \ DeLong, Good, Caldwell and Plummer \ D. M. DeLong collection; 2♂ Paratypes (OSUC), MEXICO: Iguala, Guerrero \ IX-11 39 \ D. M. DeLong collection; 1♂, 2♂ (CEAM), MEXICO: Morelos, Tetela del Volcán, San Miguel \ 1,962m \ 18°50'27.204"N, 98°44'46.895"W \ 30–Ene–2014 \ ex: *Perseaamericana* \ sweep net \ R.M. Quezada-Daniel; 1♀, 1♂ (CEAM), MEXICO, Morelos, Tetela del Volcán, Huerta El Calabazo \ 2,195m \ 18°52'3.252"N, 98°44'5.2"W \ 19–Oct–2014 \ ex: *Perseaamericana* \ sweep net. \ R. M. Quezada-Daniel; 9♂, 11♀ (CAJAPE), MEXICO: Morelos, Tetela del Volcán, San Miguel \ 1,962m \ 18°50'27.204"N, 98°44'46.895"W \ 30–Ene–2014 \ ex: *Perseaamericana* \ sweep net \ R.M. Quezada-Daniel; 1♂, 1♀ (CNIN), MEXICO: Morelos, Tetela del Volcán, San Miguel \ 1,962m \ 18°50'27.204"N, 98°44'46.895"W \ 30–Ene–2014 \ ex: *Perseaamericana* \ sweep net \ R.M. Quezada-Daniel; 1♂ (CEAM), MEXICO: Morelos, Tetela del Volcán, San Miguel \ 1,962m \ 18°50'27.204"N, 98°44'46.895"W \ 30–Ene–2014 \ ex: *Perseaamericana* \ sweep net \ R.M. Quezada-Daniel

### Key to Mexican leafhopper pest species on avocado trees (males)

**Table:** 

1	Submarginal vein of hind wing extended along apex and connected to vein R2+3 (Fig. 10)	**2**
–	Submarginal vein of hind wing not extended along apex, not connected to R2+3 or absent	**3**
2	Forewing with fourth apical cell long, slender, and parallel. Head produced and sharply angled, in lateral view, face long and strongly convex. Pronotum, mesonotum, and forewings with many tiny red spots. Aedeagus with posterior preapical processes (Fig. 13)	*** Alconeura candida ***
–	Forewing with fourth apical cell distinctly tapered distally. Head sometimes produced and angled, in lateral view, face short. Crown, pronotum, mesonotum, and forewings sometimes with orange, black or yellow marks but not red. Aedeagus with processes, if present, arising near base of shaft	**5**
3	Hind wing with apex of vein RP free, connected by crossvein to MA (Fig. 12). Crown longer than distance between eyes	*** Joruma krausi ***
–	Hind wing with RP confluent to MA, r-m crossvein absent. Crown shorter than distance between eyes	**4**
4	Pygofer with suture close to sternite VIII (Fig. 14). Aedeagus without processes	*** Empoasca angustella ***
–	Pygofer without suture close to sternite VIII. Aedeagus with pair of basal processes	*** Empoasca deskina ***
5	Inner apical cell of forewing broader basally than apically. Hind wing with three apical cells	*** Dikrella mella ***
–	Inner apical cell of forewing parallel sided. Hind wing with two apical cells	**6**
6	Pygofer process black (Fig. 15 and 16)	**7**
–	Pygofer process pale (Fig. 17)	*** Idona minuenda ***
7	Pygofer process extended beyond pygofer apex (Fig. 15)	**8**
–	Pygofer process not extended beyond pygofer, visible above dorsal margin (Fig. 16)	*** Idona gonzalezae ***
8	Clavus with small spot, not reaching margin and veins (Fig. 11)	*** Idona floresi ***
–	Clavus with large spot, reaching margin and veins (Fig. 9)	*** Idona dmitrievi ***

**Figure 9–17.**
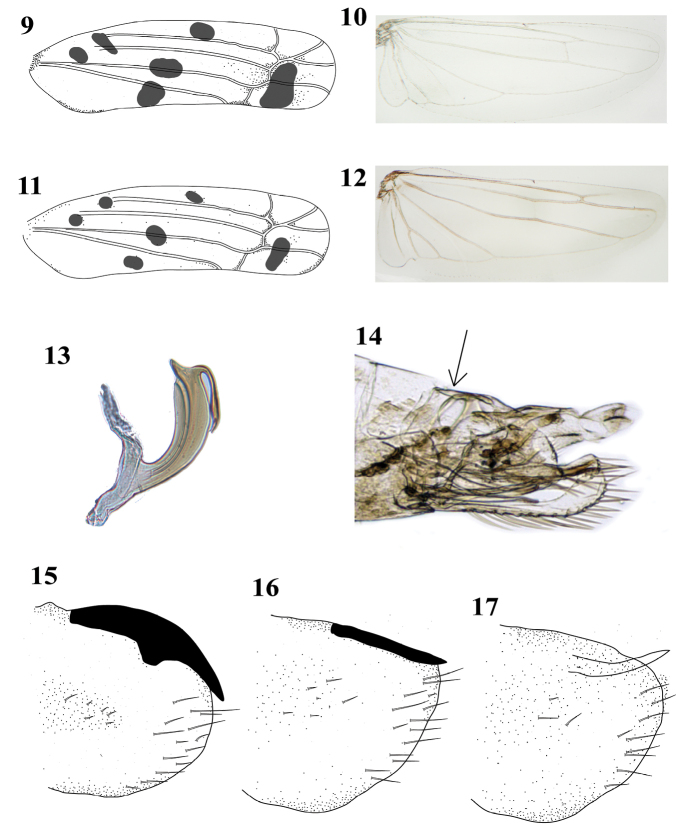
Morphological structures of microleafhopper (Typhlocybinae) species associated to avocado trees in Mexico **9** forewing of *Idonadmitrievi***10** hind wing of *Idonadmitrievi***11** forewing of *Idonafloresi***12** hind wing of *Jorumakrausi***13** aedeagus of *Alconeuracandida***14** pygofer of *Empoascaangustella*, lateral view **15** pygofer of *Idonadmitrievi*, lateral view **16** pygofer of *Idonagonzalezae*, lateral view **17** pygofer of *Idonaminuenda*, lateral view.

### Checklist of leafhoppers associated with avocado trees in Mexico

#### Alconeura (Hyloidea) candida (Ruppel & DeLong, 1952)

**Material examined.** 25♀, 2♂ (CEAM), MEXICO, Morelos, Tetela del Volcán, Huerta El Calabazo \ 2,195m \ 18°52'3.252"N, 98°44'5.2"W \ 19–Oct–2014 \ ex: *Perseaamericana* \ sweep net \ R. M. Quezada-Daniel.

#### Dikrella (Dikrella) mella Ruppel & DeLong, 1952

**Material examined.** 1♂, 2♂ (CEAM), MEXICO: Morelos, Tetela del Volcán, San Miguel \ 1,962m \ 18°50'27.204"N, 98°44'46.895"W \ 30–Ene–2014 \ ex: *Perseaamericana* \ sweep net \ R.M. Quezada-Daniel; 1♀, 1♂ (CEAM), MEXICO, Morelos, Tetela del Volcán, Huerta El Calabazo \ 2,195m \ 18°52'3.252"N, 98°44'5.2"W \ 19–Oct–2014 \ ex: *Perseaamericana* \ sweep net. \ R. M. Quezada-Daniel; 9♂, 11♀ (CAJAPE), MEXICO: Morelos, Tetela del Volcán, San Miguel \ 1,962m \ 18°50'27.204"N, 98°44'46.895"W \ 30–Ene–2014 \ ex: *Perseaamericana* \ sweep net \ R.M. Quezada-Daniel; 1♂, 1♀ (CNIN), MEXICO: Morelos, Tetela del Volcán, San Miguel \ 1,962m \ 18°50'27.204"N, 98°44'46.895"W \ 30–Ene–2014 \ ex: *Perseaamericana* \ sweep net \ R.M. Quezada-Daniel; 1♂ (CEAM), MEXICO: Morelos, Tetela del Volcán, San Miguel \ 1,962m \ 18°50'27.204"N, 98°44'46.895"W \ 30–Ene–2014 \ ex: *Perseaamericana* \ sweep net \ R.M. Quezada-Daniel

#### *Idonadmitrievi* Pinedo-Escatel & Blanco-Rodríguez, 2016

**Material examined.** 25♀, 16♂ (CEAM), MEXICO, Morelos, Tetela del Volcán, Huerta El Calabazo \ 2,195m \ 18°52'3.252"N, 98°44'5.2"W \ 19–Oct–2014 \ ex: *Perseaamericana* \ sweep net \ R. M. Quezada-Daniel; 1♂ (CAJAPE), MEXICO, Morelos, Tetela del Volcán, Huerta El Calabazo \ 2,195m \ 18°52'3.252"N, 98°44'5.2"W \ 19–Oct–2014 \ ex: *Perseaamericana* \ sweep net \ R. M. Quezada-Daniel.

#### *Idonaminuenda* (Ball, 1921)

**Material examined.** 13♀, 16♂ (CEAM), MEXICO, Morelos, Tetela del Volcán, Huerta El Calabazo \ 2,195m \ 18°52'3.252"N, 98°44'5.2"W \ 19–Oct–2014 \ ex: *Perseaamericana* \ sweep net \ R. M. Quezada-Daniel.

#### *Idonafloresi* Freytag, 2015

**Material examined.** 1♀, 1♂ (OSUC), MEXICO, Michoacán, Salvador Escalante, 03–Aug–2012 \ ex: Aguacate \ Graciela Gonzales Col.

#### *Idonagonzalezae* Freytag, 2015

**Material examined.** 1♀, 1♂ (OSUC), MEXICO, Michoacán, Salvador Escalante, 03–Aug–2012 \ ex: Aguacate \ Graciela Gonzales Col.

#### Joruma (Joruma) krausi Ruppel & DeLong, 1953

**Material type examined.** Holotype ♂ (OSUC), MEXICO: Cuernavaca Mor. \ Mexico III-1945 \ N. L. H. Krause \ D. M. DeLong collection

Additional material reviewed. 12♀, 25♂ (CEAM), MEXICO, Morelos, Tetela del Volcán, Huerta El Calabazo \ 2,195m \ 18°52'3.252"N, 98°44'5.2"W \ 19–Oct–2014 \ ex: *Perseaamericana* \ sweep net \ R. M. Quezada-Daniel.

#### Empoasca (Empoasca) deskina DeLong & Guevara, 1954

**Material examined.** 3♀, 9♂ (CEAM), MEXICO, Morelos, Tetela del Volcán, Huerta El Calabazo \ 2,195m \ 18°52'3.252"N, 98°44'5.2"W \ 19–Oct–2014 \ ex: *Perseaamericana* \ sweep net \ R. M. Quezada-Daniel.

#### Empoasca (Empoasca) angustella DeLong, 1952

**Material examined.** 6♀, 12♂ (CEAM), MEXICO, Morelos, Tetela del Volcán, Huerta El Calabazo \ 2,195m \ 18°52'3.252"N, 98°44'5.2"W \ 19–Oct–2014 \ ex: *Perseaamericana* \ sweep net \ R. M. Quezada-Daniel.

## Conclusions

Nine species in five genera of typhlocybine leafhoppers are reported from avocado trees in Mexico. None of these species have been tested or confirmed to transmit any disease so far. Species are recorded from Mexican states (Table 1), of which Morelos is the best sampled and is home to seven species. Additional sampling is underway for the purpose of management and monitoring in states with high levels of avocado production within Mexico and will undoubtedly provide additional avocado-associated records.

**Table 1.** Geographical occurrence of known leafhoppers of the subfamily Typhlocybinae inhabiting avocado trees.

**Leafhoppers**			**Distribution**
**Genus**	**Subgenus**	**Species**	**Country: state** (**known localities**)
* Alconeura *	* Hyloidea *	* candida *	Mexico: Morelos (Cuernavaca; Tetela del Volcán)
* Dikrella *	* Dikrella *	* mella *	Mexico: Morelos (Cuernavaca; Tetela del Volcán), Guerrero (Iguala), Oaxaca (Rancho Monter), and Chiapas (Vergel)
* Idona *		* dmitrievi *	Mexico: Morelos (Tetela del Volcán)
		* minuenda *	Mexico: Morelos (Tetela del Volcán), and Tamaulipas (Ciudad Victoria)
		* floresi *	Mexico: Michoacán (Uruapan)
		* gonzalezae *	Mexico: Michoacán (Uruapan)
* Joruma *	* Joruma *	* krausi *	Mexico: Morelos (Cuernavaca; Tetela del Volcán), Veracruz (Córdoba), and Oaxaca (Chiltepec)
* Empoasca *	* Empoasca *	* deskina *	Mexico: Morelos (Cuernavaca; Tetela del Volcán), Hidalgo (Jacala), Estado de México (Distrito Federal), and Veracruz (Orizaba)
		* angustella *	Mexico: Morelos (Laguna de Zempoala; Tetela del Volcán)

## Supplementary Material

XML Treatment for Dikrella

XML Treatment for Dikrella (Dikrella) mella
